# Antiangiogenic Therapy in the Treatment of Recurrent Medulloblastoma in the Adult: Case Report and Review of the Literature

**DOI:** 10.1155/2009/247873

**Published:** 2009-12-30

**Authors:** Giuseppe Privitera, Grazia Acquaviva, Giovanni Carlo Ettorre, Corrado Spatola

**Affiliations:** U.O. Radiodiagnostica e Radioterapia Oncologica, AOU Policlinico “G. Rodolico”—Catania, Via Santa Sofia 78-95125—Catania, Italy

## Abstract

Medulloblastoma is a rare tumor in central nervous system, with an even rarer occurrence in adulthood. The management of a recurrent disease is a medical challenge; chemotherapy has been used as the treatment of choice, while reirradiation has been employed in selected cases. We report the case of a 51-year-old man with recurrent medulloblastoma. He was treated with local reirradiation, chemotherapy, and antiangiogenic drug, with the latter giving the longer progression-free interval. The aim of this report is to show that recurrent medulloblastoma in adults can be approached with a multimodality treatment and that antiangiogenic therapy should have a role in the management of this disease.

## 1. Background

Medulloblastoma is a rare embryonal neuroepithelial tumor in central nervous system. It occurs most frequently in the cerebellum of children, but almost 20% of the medulloblastomas develop in adulthood. The overall frequency of medulloblastoma/PNET is very low, with the Central Brain Tumour Registry of the USA reporting that this disease is 0.9% of all reported brain tumours, with an incidence of 0.24 per 100000 person-years. The peak age group is 0–4 years, progressively declining to 0.05/10^5^ person-years for the 65- to-74 year age groups. This tumor occurs most frequently in men than women [1].

Whether medulloblastoma is the same tumor in adults and in children is an open question in the adult population.

The standard therapy for medulloblastoma has been surgical resection followed by craniospinal irradiation (CSI). The role of adjuvant chemotherapy is unclear in the adult population.

The management of a recurrent disease is a medical challenge; chemotherapy has been used as the treatment of choice, while reirradiation has been utilized in selected cases [2]. The advent of new anticancer drugs tested in brain tumors, as for antiangiogenetic molecules, has only recently been employed in the treatment of medulloblastoma and it is to be expected that their use will increase in the future, in the light of a personalized therapy. 

## 2. Case Report

We report the case of a 51-year-old man with recurrent medulloblastoma. His clinical history began in 1999, when he developed symptoms of raised intracranial pressure with dizziness and headache. A brain MR revealed a tumor in the region of right ponto-cerebellar angle, 3 cm in maximum diameter. The patient underwent a complete surgical resection, confirmed by a postsurgery MR, and a diagnosis of classic medulloblastoma, G IV WHO was performed.

He received craniospinal irradiation (36 Gy), followed by a primary boost to the posterior fossa (18 Gy) with a total dose to that region of 54 Gy.

After a 6-year period of event-free follow-up, in March 2005 a surveillance MRI showed recurrent disease in cervical and thoracic spinal cord. Thus, he received chemotherapy with dacarbazine-etoposide-cisplatin (DEC) for 6 cycles; after four cycles of this treatment a grade III-IV neurotoxicity was developed, so carboplatin was introduced in the place of cisplatin. 

In November 2005 a disease progression was diagnosed, with the evidence of recurrence in brainstem and cervical spinal cord: the patient was treated with procarbazine (60 mg/m^2^) and lomustine (110 mg/m^2^) for 2 cycles.

After that, he underwent a reirradiation limited to the sites of recurrence (brainstem and cervical spinal cord) to a total dose of 24 Gy, with concomitant temozolomide 75 mg/m^2^. Adjuvant temozolomide 200 mg/m^2^ was started after irradiation. 

**Figure 1:**
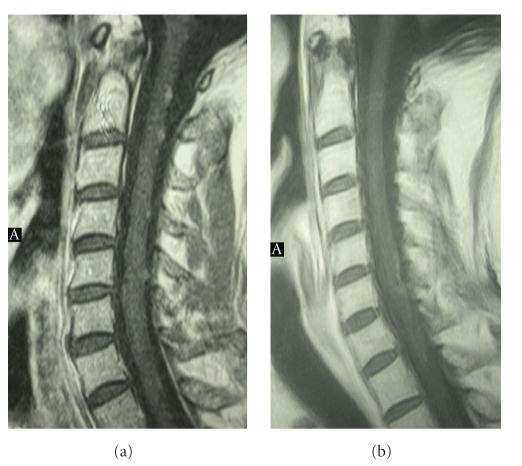
Spine MR showing the diffuse recurrent disease in the cervical spine (on the left) and the complete response during the treatment with bevacizumab (on the right).

A complete remission of the disease was demonstrated in February 2006 and, for that reason, the same treatment was continued for 13 cycles, until December 2006. During this period, the patient experienced a good quality of life and the progression-free interval was almost one year (Figure 1).

In January 2007 he had an MRI of the brain and of the spinal axis that showed recurrence in sacral spine (S1), thus we planned a new chemotherapeutic schedule, vincristine-etoposide-ifosfamide, for 2 cycles, without any evidence of response. A new control MRI evidenced a relapse in the brain and a stable disease to the spinal axis. For that reason, in view of the short relapse-free interval and of the lack of response to the conventional chemotherapeutic drugs, we decided to enter the patient into an off-label treatment with bevacizumab. Thus, in July 2007 he started this treatment with a dose of 5 mg/kg every 14 days. 

After three months on bevacizumab, the restaging MR showed a complete disease regression, without any evidence of disease in the brain and in the spinal axis. Therefore, he continued the same treatment schedule for further 4 months. During this period, the patient experienced only a mild hypertension, treated with ACE-inhibitors and a prolongation of the infusion interval, with the administration of bevacizumab at a dose of 5 mg/kg every 21 days.

In February 2008, the control MRI demonstrated a slight progressive disease in the cervical spine, consequently we returned to the previous treatment schedule of drug infusion every 14 days. After 3 months, the control MRI showed a reduction of the pathologic enhancement to the cervical spine. Thus, the treatment was continued for further 4 months until September 2008, without any relevant side effects.

In October 2008, as effect of a cranial trauma, the patient was referred to the hospital for the appearance of clinical signs of raised intracranial pressure, with sleepiness and headache, epilepsy with continuous crisis, and oliguria. A brain CT showed a subarachnoid frontal cerebral higroma, without the evidence of any vascular damage. For that reason, the patient was referred to the neurosurgeon, who performed a cerebro-spinal fluid drainage, and then to the intensive care unit for the life-saving treatments. The cytological study of the liquor was negative for cancer. During the hospitalization there was no regression of the clinical symptoms, so the patient died after 10 days from the admission (Table 1).

## 3. Discussion and Conclusions

Medulloblastoma of the cerebellum is an embryonal tumor, with a peak of incidence in children.

Not infrequently, about 20% of cases, it arises in adulthood. There is the question as to whether the pathogenesis is the same in the adult form of the disease as the childhood one [1–3]. This suggests that the prognosis and therapy of medulloblastoma could not be the same in all ages, but it is necessary to personalize the treatment approach.

The standard therapy for medulloblastoma has been surgical resection followed by craniospinal irradiation (CSI). Surgery plays a critical role in the management of this malignancy from both a diagnostic and therapeutic point of view, since the importance of complete resection is well recognized [4].

Cranio-spinal irradiation plays a key role in the management of patients with this disease for many years, because it was the only treatment available and effective, but also because of the recognition that many chemotherapy drugs have difficulties to pass through the blood-brain barrier [5]. The standard radiotherapy treatment counts a dose of 35 Gy to 36 Gy with boost to the posterior fossa, thus giving a total of 55-56 Gy with a 5-year progression-free survival (PFS) and overall survival of 50 to 65% [5, 6]. 

The role of adjuvant chemotherapy is unclear, because it was associated with a nonsignificant trend to prolonged survival. Several randomized and nonrandomised studies have demonstrated a survival benefit in pediatric medulloblastoma treated with chemotherapy, given after radiotherapy [7–9] or before it [10, 11]. Adjuvant or neoadjuvant chemotherapy plays also a key role in permitting a reduction in the dose of cranio-spinal irradiation; thus, chemotherapy is to date a standard of care in pediatric medulloblastoma, whereas its role in adult setting is not determined due to the rarity of the disease.

The management of recurrent medulloblastoma is based on the use of systemic chemotherapy. The role of reirradiation is still unclear. It has been employed in selected cases, as for patients with good performance status, longer progression-free interval, and who are not amenable to stereotactic radiotherapy. The major argument against reirradiation with fractionated external beam radiotherapy within the central nervous system is the cumulative CNS toxicity. In the recent years, brain and spinal cord reirradiation has had a reappraisal: several studies have shown a lower incidence of severe complication than previously reported [2].

The main factors determining tolerance of the CNS to irradiation seem to be total dose, interval to re-treatment, volume of brain irradiated, fraction size, use of chemotherapy, and age of patient.

There is not a gold standard chemotherapy treatment for adult medulloblastoma. Multiagent treatment with CDDP, Carboplatin, CCNU, and vincristine is the more commonly utilized treatment in high risk patients, demonstrating to increase the 5-year progression-free survival rate in children to 85 % [6, 12].

Herrlinger et al. [4] suggest that the second-line and third-line therapes should be offered to adult medulloblastoma patients. A small but significant survival benefit was demonstrated for the use of chemotherapy for high risk patients [4]. 

The novel approaches such as small molecules, monoclonal antibodies, and antiangiogenic therapies support the conventional treatment and they will increasingly allow personalized medical care.

The advent of new anticancer drugs tested in brain tumors has important consequence for personalized therapy. Tumor vasculature is emerging as an important target for antiangiogenic therapy. Slongo et al. demonstrated the expression of VEGF, VEGFR-1, and VEGFR-2 in human medulloblastoma cell lines and the possible autocrine mechanism of VEGF on medulloblastoma cell proliferation. Medulloblastoma cell lines present both VEGFR-1 and VEGFR-2. Targeting VEGF signaling may represent a new therapeutic option in the treatment of medulloblastoma [13, 14]. 

In the case presented, the patient had a first recurrence after a disease-free interval of 6 years. The patient was approached initially by means of systemic chemotherapy, with dacarbazine, etoposide and platin compounds, which results in a disease progression after 8 months, at the expenses of moderate neurotoxicity. Reirradiation was carried out after an interval of about 7 years, employing a standard fraction size and a low total dose, with concomitant and adjuvant temozolomide. It results in a partial response with a long progression-free interval of 13 months, without major side effects, with the exception of a moderate cerebral atrophy showed by the follow-up MR.

The use of systemic chemotherapy at the third relapse has not led to clinical benefit; for that reason we decided to make use of an off-label targeted therapy with an antiangiogenic molecule, such as bevacizumab. This treatment results in a rapid and complete disappearance of brain and spinal cord localizations. The only clinical relevant side effect was a moderate hypertension, which leads us to increase the interval between administration, from every 14 days to every 21 days. The every-three weeks schedule has not maintained the complete response gained by the every-two weeks schedule, so the treatment was restarted at the previous schedule, with a new antihypertensive therapy. Consequently, the follow-up MR showed again a response in the spinal cord.

During the treatment with bevacizumab, the patient experienced a good quality of life, with a progression-free interval of almost 17 months. The death was not attributable to a disease progression, neither to a treatment side effects.

The aim of this case report is to show that recurrent medulloblastoma in adults can be approached with a multimodality therapy by means of radiotherapy, chemotherapy, and targeted therapy.

In conclusion, angiogenesis seems to play a key role in the progression of medulloblastoma, and clinicians have sought to develop effective and less toxic antiangiogenic strategies, including the inhibition or destruction of abnormal blood vessels using either antiangiogenic or vascular disrupting agents [15].

## Figures and Tables

**Table 1:** Patient natural history.

Date	Treatments	Comments
April 1999	Surgery (total resection)-	DFI/6 years
	Craniospinal irradiation (35 Gy) + boost PCF (18 Gy) total dose 54 Gy	

March 2005	Relapse (cervico-dorsal spinal cord)	Progression after 8 months
	Chemotherapy (dacarbazine-vp16- CDDP/carboplatin 6 cycles)	

November 2005	Relapse (brain stem and cervical spinal cord)	Partial response PFI 13 months
	Chemotherapy (procarbazine-lomustine 2 cycles)	
	Reirradiation (brainstem and spinal cord 24 Gy) plus concomitant temozolomide	
	Temozolomide (13 cycles until December 2006)	

January 2007	Relapse (sacral spinal)	Progressive disease
	Chemotherapy (vincristine-VP16-ifosfamide-2 cycles)	

April 2007	Relapse (brain)	Complete response
	Targeted therapy (bevacizumab q.14 3 months)	

October 2007	Arterious hypertension	Progressive disease
	Targeted therapy (bevacizumab q.21 4 months)	

February 2008	Relapse (cervical spinal cord)	Partial response
	Targeted therapy (bevacizumab q.14 7 months until September 2008)	

October 2008	Cerebral hygroma/epileptic syndrome	Death
